# The League of Mercy Collections

**Published:** 1913-03-01

**Authors:** 


### The League of Mercy Collections.

### A NOTABLE INCREASE.

The following is a complete list of the collections from each district of the League for the year 1912. The grand total of £21,003 7s. 1d. shows an increase of upwards of £1,400 over the corresponding figure for 1911, viz., £19,586 10s.

### Name of Districts and Amounts, 1912.

| District | £ | s. | d. |
|---|---|---|---|
| Ashford ... ... | 100 | 17 | 5 |
| Battersea ... | 221 | 14 | 11 |
| Berkshire ... | 583 | 0 | 0 |
| Buckinghamshire | 286 | 9 | 0 |
| Bethnal Green (N.E.) ... ... | 23 | 15 | 8 |
| Biggleswade ... | 41 | 1 | 3 |
| Brentford ... | 124 | 6 | 0 |
| Brighton-Hove ... | 103 | 5 | 9 |
| Brixton ... ... | 6 | 14 | 0 |
| Camberwell (North) and Newington (West) ... ... Camberwell (Peckham) ... | 50 | 6 | 1 |
| Chelsea ... ... | 300 | 19 | 3 |
| Chertsey and Woking ... | 223 | 2 | 11 |
| Chichester ... | Nil | | |
| City of London | 538 | 1 | 2 |
| Colchester ... | 52 | 0 | 0 |
| Croydon ... | 124 | 9 | 8 |
| Dartford ... | 31 | 5 | 3 |
| Deptford-Brockley | 170 | 3 | 1 |
| Dulwich and Norwood ... ... | 175 | 7 | 9 |
| Ealing ... ... | 125 | 2 | 2 |
| Eastbourne ... | 182 | 9 | 2 |
| East Grinstead and Hayward's Heath ... ... | 97 | 19 | 2 |
| Enfield ... .. | 78 | 2 | 10 |
| Epping ... ... | 187 | 17 | 8 |
| Epsom - Esher (with Godstone-Caterham) ... | 643 | 4 | 6 |
| Essex (South-East) ... ... | 163 | 17 | 6 |
| Eye ... ... | 166 | 11 | 9 |
| Faversham ... | 38 | 4 | 0 |
| Finsbury (Central) ... ... | 6 | 6 | 0 |
| Finsbury (East) | 26 | 7 | 0 |
| Fulham ... ... | 748 | 15 | 9 |
| Gravesend ... | 53 | 15 | 6 |
| Greenwich ... | 176 | 6 | 4 |
| Guildford ... | 250 | 16 | 1 |
| Hackney (Central) ... ... | 49 | 19 | 6 |
| Hackney (North) | 103 | 18 | 6 |
| Hackney (South) | 271 | 8 | 8 |
| Hammersmith ... | 300 | 0 | 10 |
| Hampshire (North) ... | 236 | 1 | 10 |
| Hampshire (South) ... | 234 | 7 | 6 |
| Hampstead ... | 326 | 16 | 4 |
| Harrow ... ... | Nil | | |
| Harwich ... ... | 50 | 10 | 7 |
| Hertford ... ... | 173 | 7 | 6 |
| Hitchin ... ... | 127 | 2 | 8 |
| Holborn ... ... | 31 | 4 | 6 |
| Hornsey and Finchley ... | 150 | 0 | 0 |
| Horsham ... | 128 | 16 | 0 |
| Hythe ... ... | 45 | 5 | 9 |
| Isle of Thanet ... | 46 | 2 | 3 |
| Islington (West) | 58 | 19 | 6 |
| Kensington (North) ... | 356 | 5 | 8 |
| Kensington (South) ... | 1,375 | 4 | 11 |
| Kingston - Surbiton (with Wandsworth - Putney) ... | 458 | 12 | 10 |
| Lambeth (Kennington) ... | 24 | 6 | 0 |
| Lewes ... ... | 62 | 2 | 0 |
| Lewisham ... | 335 | 15 | 2 |
| Lowestoft ... | 31 | 10 | 3 |
| Luton ... ... | 227 | 1 | 11 |
| Maldon ... ... | 172 | 3 | 9 |
| Marylebone (East) ... ... | 494 | 15 | 4 |
| Marylebone (West) ... ... | 523 | 15 | 8 |
| Medway ... ... | 123 | 10 | 6 |
| Mid - Essex (Chelmsford) ... | 241 | 6 | 2 |
| Oxfordshire ... | 230 | 8 | 1 |
| Paddington (North) ... | Nil | | |
| Paddington (South) ... | 1,220 | 10 | 1 |
| Reigate ... ... | 120 | 10 | 2 |
| Richmond ... | 154 | 17 | 0 |
| Rochester ... | 18 | 10 | 0 |
| Romford and Ilford ... ... | 112 | 17 | 10 |
| Saffron Walden | Nil | | |
| St. Albans ... | 50 | 0 | 0 |
| St. George, Hanover Square ... | 1,005 | 5 | 11 |
| St. Pancras (East) | 30 | 0 | 0 |
| St. Pancras (North) and Highgate ... | 264 | 12 | 0 |
| St. Pancras (South) ... | 302 | 13 | 4 |
| St. Pancras (West) ... ... | 234 | 9 | 5 |
| Sevenoaks ... | 221 | 17 | 1 |
| Southwark (Bermondsey) ... | 100 | 15 | 0 |
| Southwark (Rotherhithe) | Nil | | |
| Southwark (West) ... ... | Nil | | |
| Stowmarket ... | 106 | 11 | 6 |
| Strand ... ... | 196 | 10 | 1 |
| Streatham ... | 75 | 15 | 6 |
| Sudbury ... ... | 42 | 13 | 0 |
| Sussex (East) ... | 330 | 9 | 2 |
| Sussex (North-East) ... ... | 211 | 17 | 10 |
| Tonbridge ... | 213 | 18 | 6 |
| Tower Hamlets (Poplar - Limehouse) ... ... | 50 | 10 | 2 |
| Tower Hamlets (Mile End) ... | 39 | 11 | 9 |
| Tower Hamlets (Whitechapel) | 65 | 12 | 0 |
| Uxbridge ... | 36 | 17 | 11 |
| Walthamstow ... | 29 | 1 | 2 |
| Watford ... ... | 80 | 0 | 0 |
| Westminster ... | 334 | 10 | 10 |
| Willesden ... | Nil | | |
| Wimbledon ... | 100 | 0 | 0 |
| Woolwich ... | 126 | 14 | 2 |
| Worthing ... | 32 | 19 | 0 |
| Total from Districts ... ... | 19,004 | 5 | 2 |
| Unallotted ... | 1,028 | 16 | 11 |
| Anonymous per Treasurer ... | 1,000 | 5 | 0 |
| Grand total of Collections for 1912 ... ... | 21,003 | 7 | 1 |

